# Young at Gut—Turning Back the Clock with the Gut Microbiome

**DOI:** 10.3390/microorganisms9030555

**Published:** 2021-03-08

**Authors:** Harish Narasimhan, Clarissa C. Ren, Sharvari Deshpande, Kristyn E. Sylvia

**Affiliations:** 1 Department of Immunology, Mayo Clinic, Rochester, MN 55905, USA; Narasimhan.Harish@mayo.edu; 2 Johns Hopkins University School of Medicine, Baltimore, MD 21205, USA; cren7@jhmi.edu; 3 Buck Institute for Research on Aging, Novato, CA 94945, USA; SDeshpande@buckinstitute.org; 4 The Society for Cardiovascular Angiography and Interventions, Washington, DC 20036, USA

**Keywords:** aging, gut–brain axis, healthspan, immune system, inflammageing, interventions, lifespan, senescence-associated secretory phenotype

## Abstract

Over the past century, we have witnessed an increase in life-expectancy due to public health measures; however, we have also seen an increase in susceptibility to chronic disease and frailty. Microbiome dysfunction may be linked to many of the conditions that increase in prevalence with age, including type 2 diabetes, cardiovascular disease, Alzheimer’s disease, and cancer, suggesting the need for further research on these connections. Moreover, because both non-modifiable (e.g., age, sex, genetics) and environmental (e.g., diet, infection) factors can influence the microbiome, there are vast opportunities for the use of interventions related to the microbiome to promote lifespan and healthspan in aging populations. To understand the mechanisms mediating many of the interventions discussed in this review, we also provide an overview of the gut microbiome’s relationships with the immune system, aging, and the brain. Importantly, we explore how inflammageing (low-grade chronic inflammation that often develops with age), systemic inflammation, and senescent cells may arise from and relate to the gut microbiome. Furthermore, we explore in detail the complex gut–brain axis and the evidence surrounding how gut dysbiosis may be implicated in several age-associated neurodegenerative diseases. We also examine current research on potential interventions for healthspan and lifespan as they relate to the changes taking place in the microbiome during aging; and we begin to explore how the reduction in senescent cells and senescence-associated secretory phenotype (SASP) interplay with the microbiome during the aging process and highlight avenues for further research in this area.

## 1. Introduction

The microbiota, which is a dynamic community of bacteria, fungi, viruses, archaea, and microbial eukaryotes, is important to both lifespan and healthspan. While lifespan refers to the number of years lived, healthspan considers the quality of life and length of life that is disease-free and functional [1]. Over the past century, humans have increased their life-expectancy by roughly 30 years due to public health measures, such as clean water and sanitation [1]. However, with increasing age also comes increasing susceptibility to chronic disease and frailty [2,3]. Dysfunction of the microbiome can be linked to many conditions that increase in prevalence with aging, including type 2 diabetes, cardiovascular disease, Alzheimer’s disease (AD), and cancer [4,5,6,7].

When the community of various bacteria and other microbes are balanced, coexist peacefully, and benefit the health of the host, the microbiome is said to be in a state of “eubiosis” [8]. This state in healthy individuals is typically composed of five predominant phyla: Firmicutes (79.4%), Bacteroidetes (16.9%), Actinobacteria (2.5%), Proteobacteria (1%), and Verrumicrobia (0.1%) [8,9]. However, the gut microbiota is also an everchanging entity, influenced by many factors including age.

The microbiome exhibits a continuous aging process alongside normal host aging [10,11]. Gut microbial communities of children, adults, the elderly, and centenarians have been found to cluster apart from one another other [12,13], suggesting that the gut microbial communities may in fact play different roles throughout the aging process. The development of age-related diseases is often associated with gut dysbiosis, changes in the gut microbiota that negatively impact host health [14,15]. Furthermore, aging is typically associated with decreased diversity and Firmicutes to Bacteroides ratio, as well as an increase in some Proteobacteria, opportunistic species, and pathobionts [12,16,17,18,19,20,21].

Interestingly, centenarians and semi-supercentenarians, studied as models for longevity, have a higher abundance of microbes in their gut associated with health, including *Akkermansia*, *Christensenellaceae*, *Bifidobacterium*, and *Lactobacillus* spp., compared to young adults and/or the elderly [16,22,23,24,25,26]. *Akkermansia*, *Bifidobacterium*, and *Lactobacillus* have all been shown to reduce inflammation and maintain intestinal integrity, suggesting an integral role of the immune system in aging [27,28,29,30]. Further, *Christensenallaceae* is associated with low body mass index (BMI) and is linked to host genetics, indicating its potential role in heredity and longevity as well [31]. Some studies indicate that long-living individuals also have greater alpha diversity in their gut microbiome [18,25], while others suggest that alpha-diversity is lower in centenarians [32]. Sufficient diversity protects against environmental challenges, while low gut microbiome diversity is associated with frailty, obesity, inflammatory bowel disease (IBD), cancer, type 2 diabetes, and autoimmune diseases [10,15,33]. However, centenarians can also have lower abundance of beneficial bacteria in their gut, including *Faecalibacterium* [18], so although the microbiome appears to play a role in healthy aging, more research is necessary to determine if there is a causal relationship.

Many factors can influence the microbiome, including non-modifiable factors like age, sex, and genetics [31,34,35,36]. For example, monozygotic twins have more similar gut microbiotas than dizygotic twins [31]. However, modifiable factors, like many environmental contributors, including diet, medication, and infections, can also contribute to the microbiome [37,38,39,40]. For example, the shift from plant-based to animal-based diets increase bile-tolerant microbes and decrease the abundance of Firmicutes that help to metabolize plant polysaccharides [39]. Many of these modifiable factors thus provide useful opportunities for intervention to promote lifespan and healthspan in aging populations.

To understand the mechanisms behind these interventions during aging, it can be helpful to understand the gut microbiome’s bidirectional relationship with the immune system. Low-grade chronic inflammation tends to develop with age, a phenomenon termed “inflammageing” [41]. Systemic inflammation, a feature of inflammageing, stems from the gut microbiome as well as senescent cells. Senescent cells are cells that stop dividing, accumulate with age, and adversely affect the microenvironment through secretion of pro-inflammatory modulators including cytokines, chemokines, proteases, growth factors, and bioactive lipids, which in turn impact the gut microbiome [42,43]. The resulting microenvironment from these secretions is termed the senescence-associated secretory phenotype (SASP).

Furthermore, the significance of the bidirectional communication between the brain and gut, termed the gut–brain axis (GBA) has become well-established over recent years. The crosstalk between the complex system consisting of the nervous system (central, autonomic, and enteric), the gut microbiota, and the hypothalamic pituitary adrenal (HPA) axis is regulated by neuro-immuno-metabolic-endocrine mediators [44]. Although the importance of the microbiome in the GBA is being increasingly appreciated, dysbiosis has also been implicated in several age-associated neurodegenerative diseases [45] with markedly limited evidence compared to other areas of study.

**Figure 1:**
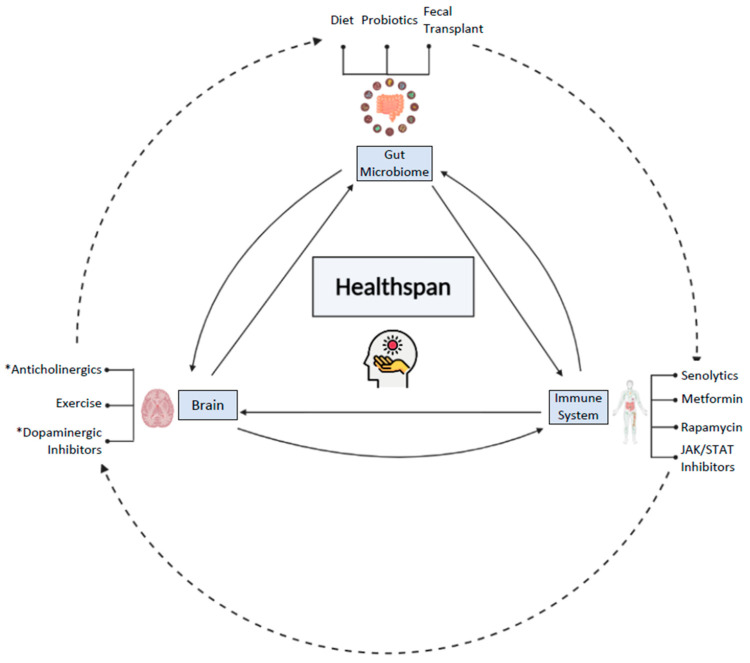
The gut microbiome, immune system, and brain all cross-communicate and can be modulated by interventions to improve healthspan and lifespan. Some interventions (Section 4) may more directly impact one system, some may impact indirectly, such as the direct impact of diet (Section 4.2) and fecal transplant (Section 4.5), and the indirect impact of exercise (Section 4.8) on the gut microbiome. Nonetheless, the gut microbiome (Section 4.2, Section 4.3, Section 4.4 and Section 4.5), the brain (Section 2 and Section 4.8), and the immune system (Section 4.6 and Section 4.7) are all connected to one another, and along with age, change in one system can subsequently impact the other systems, ultimately impacting healthspan and lifespan as well. Moreover, the impact of individuals and combinations of interventions on the key players of healthspan and lifespan are still being explored (Section 5). (*) indicates that a drug more specifically improves healthspan for Alzheimer’s/Parkinson’s patients, whereas the other interventions are meant for a broader population. This figure was created with Biorender.com.

The way in which the microbiome changes with age and how the immune system and the GBA work together to mediate changes across the lifespan and healthspan are not well understood; however, interventions targeting diseases and disorders of aging may be better understood by a thorough understanding of the mechanisms underlying the aging microbiome. In this review, we examine how the changes in the microbiome with age are modulated by the immune system and GBA along with interventions for healthspan and lifespan, and begin to explore how the SASP and microbiome might be interwoven during the aging process (Figure 1).

## 2. The Gut–Brain Axis and Aging

The gut microbiome influences central nervous system (CNS) homeostasis via a variety of mechanisms that can have subsequent effects on lifespan and healthspan. Certain species of commensal bacteria are capable of secreting neurotransmitters (e.g., Gamma-amino butyric acid (GABA), noradrenaline, and dopamine) and altering host production of neurotransmitters in the body [46]. Short chain fatty acids (SCFAs) produced by bacterial fermentation of dietary fibers and resistant starches are known to possess neuroactive and/or epigenetic properties, playing a role in neuro-immunoendocrine homeostasis [47]. Following production of SCFAs, studies have shown that SCFAs can also influence blood–brain barrier (BBB) integrity, with germ-free (GF) mice exhibiting increased BBB permeability [48]. Furthermore, SCFAs can also traverse and modulate neurotrophic factor levels, neurogenesis, and neuroinflammation, thus contributing to neuronal function [47]. Additional neuroactive metabolites, such as kynurenine and its derivatives produced via tryptophan metabolism by the gut microbiome, have also been shown to regulate neuronal activity in the CNS and the periphery [49].

Intestinal immunity is also affected by the presence and activity of the gut microbiome. Activation of receptors, such as Toll-like receptors and Nod-like receptors on innate immune cells, as well as regulation of T helper 17 cells (pro-inflammatory) and regulatory T-cells (anti-inflammatory), influence intestinal immune homeostasis [50]. Beyond exerting immunomodulatory effects within the gastrointestinal (GI) tract, peripheral responses associated with CNS disorders have been associated with gut microbiota dysbiosis [51]. Low grade systemic inflammation has been observed in several neuropsychiatric disorders, with some studies attributing this phenomenon to translocation of microbial components from the gut [51].

Several of the aforementioned effects exerted by the gut microbiome were discovered using GF animals, which exhibit prominent deficits in the development and functioning of the enteric nervous system (ENS) and CNS [52]. In addition to notable changes in gut sensory-motor functions and neuromuscular function, drastic changes in stress responses, anxiety, and depression-like behavior have been observed in GF mice as well [52].

Various model systems have helped to understand that AD is characterized by the presence of plaques composed of beta (β)-amyloid fibrils and hyperphosphorylated tau protein in several parts of the brain. Numerous studies have revealed an association between intestinal health (gut dysbiosis in particular) and the aggregation of amyloid (A)β peptides in intestinal epithelial cells [53]. Various species of the gut microbiota secrete a mixture of amyloid species along with lipopolysaccharide (LPS), which are capable of polymerization and forming insoluble aggregates [54]. Bacterial amyloid proteins also bear significant structural similarity to human Aβ, potentially inducing an immune response against self-antigens via molecular mimicry [55]. Antigens derived from bacteria are also capable of translocation into the CNS to trigger an inflammatory response, evidenced by the presence of elevated levels of LPS and E. coli pili in the brains of AD patients [56]. Responses to other bacterial components in a “leaky” aging gut also induce a systemic inflammatory state resulting in damage, including within the CNS [56,57]. In a similar fashion, Gram-negative bacteria on the dental biofilm may also be able to infiltrate the brain in pathological conditions like periodontitis. Several studies have observed an association between periodontitis and the onset of AD, implicating the “leaky gum” in promoting toll-like receptor 4 (TLR-4) and TLR-2 mediated inflammation [58]. A positive correlation between Aβ load in the brain and periodontal disease (cytokine levels as well as IgG titers) further strengthens this hypothesis, reiterating the role of peripheral inflammation in AD [59,60]. In spite of numerous correlational studies in humans, a causal association between periodontitis and AD is yet to be established [58,61].

An alternative hypothesis suggests the accumulation of orally transmitted prions within intestinal secondary lymphoid tissues, such as Peyer’s patches and mesenteric lymph nodes, resulting in the transport and propagation of prionopathies to the CNS [62]. Additionally, insulin resistance has recently been shown to develop in the brains of those with AD, with impaired insulin signaling observed in non-diabetic patients as well [63]. As alterations in the gut microbiome have been shown to regulate brain insulin sensitivity, it is possible that AD-associated changes in the microbiome result in insulin resistance within the brain [63]. Although a definitive mechanism of action is still unknown, gut microbiome dysbiosis may contribute to AD pathogenesis via a combination of increased amyloid production, activation of peripheral and CNS immune responses, as well as insulin resistance.

Furthermore, Parkinson’s Disease (PD) is a result of progressive dopaminergic neuronal death in the substania nigra, leading to a loss of dopaminergic signaling. Some PD patients have abnormal accumulation of alpha-synuclein (αSYN), forming Lewy bodies; and numerous studies suggest that αSYN aggregates in the intestines of healthy individuals who later develop PD [62,64]. Furthermore, increased intestinal permeability is also observed in PD patients, further suggesting the potential role of translocated gut microbial components in inducing systemic and neuroinflammation [57]. A significant decrease in *Prevotella* spp. and *Clostridium* spp. has been observed in patients suffering from PD; however, the exact role of these bacterial populations in pathogenesis is still not understood [65]. Based on current evidence, the intestinal microbiome is thought to promote αSYN transport to the CNS as well as aggregation via neuroinflammation. Establishment of a causal link between dysbiosis of specific populations of the microbiome and PD would pave the way for the development of biomarkers for early diagnosis as well as targets for intervention.

The role of the GBA has been studied in several other age-associated conditions including amyotrophic lateral sclerosis (ALS), stroke, and cognitive decline [66,67]. While different mechanisms have been hypothesized to mediate dysfunction in these conditions, the aged microbiome and associated changes are an often-overlooked commonality. Several other poorly understood mechanisms may be at play in affected CNS function and subsequent dysfunction, prompting the need for more research. Polymorphisms in genes such as the fat mass and obesity-associated gene (*Fto*) are known to impact brain function, with recent studies indicating a potential role of the gut microbiome in this process [68,69]. In a mouse model of ALS, a pro-inflammatory gut microbiome dictated the severity of disease and lifespan rather than the mere presence of the common ALS genetic mutation [70]. Since a low-grade inflammatory state is observed in both aging as well as neurodegenerative diseases, this phenotype may (in part) be driven by the aged microbiome.

The population and diversity of the gut microbiome has been shown to change drastically with age, presumably due to numerous physiological changes, including alterations in chemical composition and structure of the colon. As aging is the most prominent risk factor for the development of several neurodegenerative diseases and other neurological conditions, the role of the aged microbiome in pathogenesis warrants further investigation. However, such studies have proven to be highly challenging due to variance in the abundance of families (one levelling another) within a phylum, resulting in an inability to detect significant changes at a low-taxonomic resolution [7]. The identification of specific changes at the genus/species level would be critical to reveal the precise contribution of microbial populations to pathogenesis of neurodegenerative diseases. However, with a large cohort of patients, it may be possible to identify the specific gut microbial populations associated with disease. Such information would facilitate an insight into the mechanisms employed by the aged microbiome, which is critical for the development of diagnostics and therapeutic interventions to mitigate the adverse effects of aging on the CNS.

## 3. The Gut Microbiome and Inflammageing

The gut microbiome exerts a profound effect on both local and systemic immune responses [71,72]. Having co-evolved with the gut microbiota, immune cells have developed several methods to maintain tolerance toward these commensal populations [73]. High levels of immunosuppressive cytokines, such as interleukin (IL)-10 and transforming growth factor beta (TGFb), along with regulatory T-cells and tolerogenic macrophages, ensure a non-inflammatory profile of resident immune cells under homeostasis [73,74]. However, upon dysbiosis or increased permeability of intestines, an inflammatory response is induced in the gut and often at a systemic level, contributing to the development of disease.

Increased translocation of bacterial components, such as LPS due to compromised intestinal barrier integrity, has been shown to trigger a TLR4 mediated pro-inflammatory response in the gut as well as distant regions [41]. Elevated levels of LPS have been observed in the plasma and feces of aged mice, with a concomitant induction of nuclear factor kappa beta (NF-kB) in the colon, possibly contributing to “inflammageing” [41]. The increased intestinal permeability observed with aging was originally thought to occur due to a reduction in the secretion of mucin as well as expression of tight junction proteins. However, a recent human study demonstrated a negligible difference in permeability with aging in healthy subjects suggesting alternative mechanisms in mediating heightened systemic inflammation [75].

Furthermore, age-associated elevation of inflammatory mediators in circulation is abrogated in GF mice, implicating the gut microbiome specifically in inflammageing [76]. Additionally, co-housing GF mice with old but not young conventional mice results in an increase in circulating pro-inflammatory cytokines [76]. In humans, IL-6 in particular, is elevated in the elderly who exhibit reduced barrier integrity [77]. IL-6 is known to play a critical role in promoting differentiation of T-cells to pro-inflammatory Th17 cells over immunosuppressive Tregs, potentially contributing to inflammageing. The presence of increased circulating levels of Th17 cells in the aged supports this hypothesis [78].

SCFAs released by gut bacteria following metabolism of complex carbohydrates are also capable of modulating inflammatory responses. Microbiota-derived SCFAs promote antigen-specific Th1 cell IL-10 production via G-protein coupled receptor (GPCR) signaling, facilitating a tolerogenic environment in the gut [79]. Butyrate, a known histone deacetylase inhibitor, impedes pro-inflammatory pathways from acting via epigenetic interactions as well as by direct binding to GPCRs, inducing Treg development [80]. IBD patients suffering from dysregulated inflammatory responses in the colon also exhibit a reduction in butyrate-producing bacteria, such as *Faecalibacterium prausnitzii* [81]. This bacterial population is known to decrease with age, possibly contributing to diminished anti-inflammatory responses in the gut during aging.

The aged gut microbiome is directly implicated in systemic inflammation (feature of inflammageing) via a fecal microbiota transfer from young or aged mice to young GF mice [82]. Specifically, only aged microbiota results in enhanced CD4+ T-cell differentiation in recipient spleens, suggesting a systemic effect of the transfer. Furthermore, higher pathogen recognition receptor (PRR) activity in serum as well as upregulation of TNF-α in the ileum is observed in these mice. Elevated PRR activity, particularly TLR2 and TLR4, indicates the presence of increased levels of bacterial components in circulation [82]. Increased IL-6, TNF-α, eotaxin, and RANTES are observed following stroke in young mice with an aged microbiota compared to those with young microbiota [83], reiterating once more the role of the aged microbiome in systemic inflammation. While additional studies are required to implicate specific microbial populations, the aged microbiota appears to be capable of inducing some features of inflammageing. Confirming findings from rodent models, age-associated increases in IL-6 and IL-8 is linked to an increase in Proteobacteria and a concomitant reduction in *Ruminococcus lactaris et rel*, suggesting that specific age-related changes in the composition of microbiota may be driving inflammageing [16].

Dysfunctional macrophage activity is also driven by age-associated elevation of TNF-α [76]. Chronic exposure to TNF-α results in impaired macrophage anti-bacterial activity against *S. pneumoniae*, consistent with clinical data in which older individuals are at increased risk of developing and dying from *S. pneumoniae* infection [76,84]. The role of the gut microbiome in mediating this phenotype has also been confirmed in aged GF mice that not only lived 600 days longer than conventional mice, raising important questions about the microbiome and lifespan, but were also protected from age-associated inflammation [76]. Furthermore, macrophages derived from these GF mice efficiently cleared *S. pneumonia* while their specific-pathogen-free (SPF) age-matched controls could not [76].

Given the myriad mechanisms by which the gut microbiome appears to affect as well as mediate the immunological effects of aging, it is imperative to improve our understanding of these mechanisms. Firstly, it is essential to confirm whether age-associated gut dysbiosis is yet another consequence or a driver of inflammageing. Insight into these interactions would pave the way for gut-targeted therapies to either alleviate features of inflammageing or, more excitingly, delay aging itself. Since immune dysfunction is implicated in several aspects of aging and associated pathologies including neurodegenerative disease, the potential for such interventions restoring immune homeostasis is immense.

## 4. Interventions

Although life expectancy has increased over the past century, frailty and disease susceptibility have also increased along with increasing age. Significant advancements have been made in anti-aging medicine (e.g., calorie restriction (CR), senolytics), gut microbiome therapeutics (e.g., probiotics, fecal transplants), and therapeutics for neurodegenerative diseases (e.g., exercise, pharmacological therapies) in the last few decades to target some of the challenges of living longer, with more research on the horizon [83]. Although these interventions are often categorized separately, they are all interconnected, and it may be important to treat them as such as we move forward in the field.

### 4.1. Calorie Restriction

CR, which involves reduced caloric intake (typically 30–40%), without malnutrition or loss of essential nutrients, extends healthspan and lifespan in diverse models including rodents and primates, and impedes the accumulation of molecular damage [85]. CR can help to extend healthspan and lifespan via various mechanisms. For example, it can help prevent accumulation of cellular senescence through decreasing oxidative stress and inflammation, increasing sirtuins (which also helps reduce oxidative stress), increasing autophagy, enhancing DNA repair mechanisms, and inhibiting IGF-1 and mTOR [85,86]. CR also helps prevent neurodegenerative diseases through increasing neurogenesis, synaptic plasticity, and neuroprotection [87]. Evidence for how the gut microbiome plays a role in CR’s contribution to longevity is also emerging [88,89,90,91].

In mice, CR can help to mitigate age-related shifts in the gut microbiome, such as through increasing the relative abundance of *Lactobacillus*, *Bifidobacterium*, *Akkermansia*, *Allobaculum*, and *Parasutterella* [89] or through reducing age-related increases in Bacteroides and decreases in numerous genera within the Firmicutes phylum [92]. While some studies have found that CR affects the Firmicutes or Bacteroidetes phylum, and/or the Firmicutes/Bacteroidetes ratio in either direction [89,92,93,94], others have found no such changes [95]. There are variations in the CR shifts in the gut microbiome, likely due to the high variation between study models and design (e.g., length of CR), suggesting the need for consistency in the length of CR intervention sufficient to affect change, as well as considering lab methods, storage conditions, and amplification method [96].

### 4.2. Diet

Diet plays a critical role, not only in healthspan and lifespan [97], but also in maintaining an optimal gut microbiome composition [98]. The Nu-AGE study found that in healthy older adults, plant-based diets (e.g., Mediterranean diet) are associated with increased alpha-diversity, increased relative abundance of the Bacteroidetes taxa, and increased anti-inflammatory microbials, including *Faecalibacterium prausnitzii*, *Eubacterium rectale*, and *E. biforme*; however, animal-based diets are associated with pro-inflammatory microbes such as *Ruminococcus gnavus* and *Collinsella* spp. [99]. To combat inflammation and the decreased SCFA production often seen in the elderly, an adequate amount of fiber should also be consumed [100,101,102,103,104]. Most prebiotics, which promote the growth of intestinal microbes, are associated with health and wellbeing and can be classified as dietary fibers [105]. The data from the Nu-AGE study demonstrate that diets like the Mediterranean diet can help curb the onset of frailty through preserving gut microbial diversity and decreasing inflammation.

### 4.3. Probiotics

Probiotics, which are live microorganisms that in appropriate amounts can benefit host health [106], are emerging as an intervention to promote lifespan and healthspan in model organisms. Probiotics commonly consist of *Lactobacillus* spp. or *Bifidobacterium* spp., which are both correlated strongly with lifespan [91,93,95,107,108,109,110,111]. *Lactobacillus* spp. prolongs C. elegans’ lifespan by about 10–20% [22,112,113] by altering the DAF-16/insulin-like pathway [112], increasing resilience to oxidative stress [110], stimulating the innate immune response [114], and mediating nuclear hormone receptors and PMK-1 signaling [115]. In aging rats, probiotics modulate AMPK activity and prevent telomere shortening [116]; protect aging bone and muscle [117]; and improve lipid, renal, and liver profiles, while also inhibiting the growth of pathogens *Escherichia coli*, *Staphylococcus aureus*, and *Staphylococcus epidermidis* [118]. However, in wild-type Drosophila melanogaster, although a probiotic mixture increases mean and median lifespan, it decreases mean and median lifespan in Drosophila melanogaster with insulin receptor deficiency and a type 2 diabetes mellitus phenotype [119]. These preliminary studies suggest that although probiotics may impact pathways involved in longevity, they may not universally prolong lifespan, and thus, more work is required to distinguish factors associated with the utility or possible harm of probiotics.

Modulating the composition of the gut microbiome using probiotics can also rescue the function of several immune cells as well as improve the inflammatory state of the gut. For example, a mixture of *Bifidobacterium longum* and *Lactobacillus helveticus* boosts levels of regulatory T and B-cells in aged individuals [120]. Furthermore, Bacillus coagulans can increase production of anti-inflammatory cytokines in aged humans, thus resulting in an increase in *Faecalibacterium prausnitzii* populations [121]. Interestingly, one study showed that a probiotic mixture of several gram-positive bacterial strains results in several changes in the microbiome, including an increase in Actinobacteria and Bacteriodete in aged mice [122]. These changes alter the expression of several inflammatory and neuronal plasticity genes in the brain reiterating the relevance of the GBA as well. Supplementation with *Lactobacillus rhamnosus* can also restore the Th1/Th2 balance in aged mice, another phenomenon typically observed in inflammageing [123]. *Lactobacillus* spp. can not only mitigate inflammageing, but they also modulate SASP, such as modulating the products secreted by adipose tissue and adipokines [124]. Furthermore, administering *Lactobacillus fermentum* to H_2_O_2_-induced cellular senescence in preadipocytes can mitigate senescence markers [125]. These studies provide support that modulation of immune responses via direct changes in gut microbial populations or associated metabolites could be immensely useful tools, particularly in the aged where immune homeostasis is significantly disrupted. Further research is critical to understand the role of specific microbial species needed to maintain homeostasis to identify relevant therapeutic targets and alleviate inflammageing and SASP.

Probiotics also interact with the GBA and have been explored as a novel therapeutic for neurodegenerative diseases. The effects of probiotics on inflammageing and oxidative stress are involved in the pathogenesis of neurodegeneration [126]. Furthermore, *Lactobacillus* spp. and *Bifidobacterium* [127] both inhibit pathogen disruption of the gut barrier, which could suggest protection against age-related gut neurodegeneration [126,128]. Further data suggest that probiotics may in fact aid in mental abilities and metabolism. For example, in a randomized, double-blind, controlled clinical trial of 60 AD patients, the probiotic group showed better mini-mental status exam scores and metabolic measures after 12 weeks compared to the control group [129]. Evidence from clinical trials showing the impact of probiotics in neurodegenerative diseases and aging are still scarce and often inconsistent; therefore, further investigations are needed to determine which probiotics in which doses will benefit which particular disease state and in what populations.

### 4.4. Prebiotics

Prebiotics are substrates, including food and non-food, that the host microorganism can utilize for a health benefit [8,130]. When prebiotics are combined with probiotics, it is termed “synbiotics.” In a randomized controlled trial of aging adults older than 65 years, prebiotic administration improved exhaustion and handgrip strength (criteria for frailty), likely through influencing the gut microbiota–muscle–brain axis [131]. However, prebiotic administration did not significantly affect the overall rate of frailty [131]. In another study, prebiotics, along with probiotics and synbiotics, influenced serum calcium levels and therefore benefited bone health in aging adults, but did not impact bone mineral density, parathyroid hormone, osteocalcin, nor alkaline phosophatase [132]. Therefore, prebiotics show potential in improving healthspan independent of probiotics, although there may still be limitations to the use of prebiotics.

### 4.5. Fecal Microbiota Transplants

A fecal microbiota transplant (FMT), which involves transferring donor’s stool to the GI tract of the recipient to change the recipient’s gut microbiome, shows potential for anti-aging. FMT is the current accepted treatment for recurrent *Clostridium* (*C*.) *difficile* infection in adults, and has successfully alleviated abdominal pain, bloating, and diarrhea, while also increasing microbiota diversity in elderly patients infected with *C. difficile* [133]. In a recent study, progeroid mice (showing intestinal dysbiosis along with neurodegeneration) received FMT from wild-type (WT) mice and supplementation with *Akkermansia muciniphila*. FMT and supplementation increased both lifespan and healthspan by restoring secondary bile acids, suggesting the role of the gut in maintaining health [32]. Similarly, transplanting microbiota from long-living humans into mice results in greater alpha diversity, increased *Lactobacillus* and *Bifidobacterium* (probiotic genera), increased *Roseburia*, *Faecalibacterium*, *Ruminococcus*, *Coprococcus* (SCFA producing genera), and decreased lipofuscin and β-galactosidase accumulation in the gut microbiome, all suggesting potential benefits to lifespan and healthspan [134].

Furthermore, microbiome transplants from healthy WT mice to transgenic mice with AD-like pathology alleviates the formation of amyloid plaques and neurofibrillary tangles and mitigates expression of genes for intestinal macrophage activity and circulating blood inflammatory monocytes [135]. Additionally, FMT from WT mice to transgenic mice decreases deposition of amyloid-β, cyclooxygenase-2 (COX-2), and cluster of differentiation 11b (CD11b) levels, increases synaptic plasticity, and reverses gut microbiota and SCFAs changes [136]. These data and others [137] all suggest that FMT may help to counteract aging-associated neurodegenerative changes through interactions with the immune system and cognitive-preserving effects.

### 4.6. Antagonism of SASP: Metformin, Rapamycin, and JAK-STAT Inhibitors

One strategy for fighting cellular senescence is pharmacological therapy that can decrease the amount of SASP molecules produced by already present senescent cells that mitigate deleterious effects. The challenge with this action, however, is ensuring that the anti-oncogenic properties do not decrease [43,138]. These pharmacological therapies include metformin, rapamycin, and JAK-STAT inhibitors, and they may not only help with anti-aging through SASP, but also may interact with the gut microbiome.

Metformin is an antidiabetic and antihyperglycemic medication that works by activating AMP protein kinase, while also blocking SASP in transformed fibroblasts and increasing healthspan and lifespan [139,140]. Metformin can also alter gut microbiome composition, counteracting age-related changes through inhibiting Firmicutes, and increasing the relative abundance of *Akkermansia*, bifidobacterial species, and SCFA-producing species [140,141,142,143]. Beneficial modulation of the gut microbiome through metformin increases goblet cell mass and mucin production in older mice, which protects against aging-related leaky gut, inflammation, and cognitive decline [144]. Finally, increased *Escherichia* spp. in metformin users helps explain the decreased risk of hospitalization for infective diseases [144].

Additionally, Rapamycin (sirolimus) is an immunosuppressant drug that binds to the mammalian target rapamycin (mTOR), thus decreasing transcription of some SASP products and increasing autophagy of senescent cells [145,146]. Six weeks of rapamycin can improve healthspan measures in mice, with associated shifts in the microbiome toward increased segmented filamentous bacteria in the small intestine [145]. Furthermore, in Drosophila, although treatment with rapamycin may alter the gut microbiome, modulation of the gut microbiome through its use may not play a direct role in increasing lifespan given that elimination of the microbiota still results in the anti-aging effects of the drug [147].

Finally, JAK-STAT inhibitors hold the potential for regulating the impact of aging because of the JAK-STAT signaling pathway’s connection to proinflammatory products [148,149]. Inhibition of JAK-STAT signaling extends the lifespan of Drosophila through preventing metaplasia/gut proliferation and microbiota dysbiosis [150,151], and therefore, holds great potential in therapeutic intervention in humans as well. Although little research has been done to date investigating the effect of JAK-STAT inhibitors on the gut microbiome in humans, the evidence thus far holds potential [152].

### 4.7. Deletion of Senescent Cells: Senolytics

Cellular senescence is a double-edged sword [153]. What constitutes an anticancer mechanism in early life exhibits tumor-promoting activities at a later stage, a phenomenon called Antagonistic Pleiotropy [154]. A rational solution to this phenomenon is deletion of these senescent cells in aging individuals. The Mayo Clinic pioneered work in developing “Senolytics” largely by targeting pro-survival pathways, such as Bcl-2 [155]. There are currently many compounds, both naturally occurring and manufactured drugs, being evaluated for their senolytic activity. Yousefzadeh et al. tested various flavonoids, out of which fisetin has yielded good senolytic activity, and luteolin and curcumin displayed weak, yet senolytic properties [156]. Some of these senolytic flavonoids, interestingly, have also been shown to affect the microbiota.

Quercetin, a plant-derived polyphenol, is shown to have anti-carcinogenic as well as antiviral activities and several other beneficial effects on inflammation and the immune system [157]. When consumed as a dietary supplement, it augments the microbial population of Bacteroides, Bifidobacterium, *Lactobaccilus*, and *Clostridia*. It also reduces *C. rodentium*-induced colitis and associated pro-inflammatory cytokines [158]. Fisetin, a flavonoid found in a numerous commonly consumed fruits and vegetables, can affect the diversity and distribution of the gut microbiota in a premature ovarian failure (POF) mouse model. The fistein-induced increase in bacterium *Lachnospiraceae* is also associated with a decrease in the CCR9+/CXCR3+/CD4+ T lymphocytes in blood [159]. Furthermore, oral ingestion of Curcumin, a major constituent of turmeric and heavily recognized for its medicinal benefits, has been shown to affect the diversity and composition of the gut microbiota [160,161,162].

Aspirin, consumed by around one-fifth of adults in the United States, has been associated with senolytic characteristics as well [163,164]. A 2019 randomized placebo-controlled trial assessed the impact of daily Aspirin consumption over 4–6 weeks in older adults. Subjects receiving Aspirin had decreased abundance of *Parabacteroides* and *Dorea*, (often increased in cases of colorectal cancer). Furthermore, *Akkermansia* (associated with anti-cancer activity) increased in those who took Aspirin compared to those who received a placebo [165].

While there is some evidence for the influence these naturally occurring senolytics have on the microbiome, there is a paucity of research on impact of synthetics senolytics on the microbiome. With the emergence of clinical trials in developing senolytics drugs safe for human consumption, such as Dasatinib and Unity Biotechnology’s candidate UBX101, the drugs’ interplay with the aging microbiome are important elements to study on the aging population around the country.

### 4.8. Therapeutics for Neurodegenerative Diseases

Multiple randomized control trials have demonstrated the neuroprotective role of exercise, which alleviates neuropsychiatric symptoms and functional decline [166,167,168], and like diet, exercise can significantly shift the gut microbiome composition and improve healthy aging. Exercise increases the proportion of *Bifidobacterium* [169], the Bacteroidetes: Firmicutes ratio [170], or increases the abundance of *Akkermansia* and *Butyricimonas* in mice [169,170]; and in recent human studies, female and male lean participants who exercise exhibit higher SCFAs [171,172]; and active women show increased *Akkermansia*, *Faecalibacterium prausnitzii*, and *Roseburia hominis* [173]. Furthermore, exercise likely plays a role in the GBA through vagus nerve tension, which helps with anti-inflammatory regulation [174,175], though further work is needed in this area of research.

Interestingly, various pharmacotherapies are used to treat neurodegenerative diseases, including cholinesterase inhibitors, memantine, and vitamin E for AD [176], as well as dopaminergic medications (carbidopa/levodopa), COMT-inhibitors, and anticholinergics for Parkinson’s disease [177,178]. Cholinesterase inhibitors and memantine are currently the only symptomatic medications for AD patients, but although these medications can help with cognition and global functioning, there is little evidence that these pharmacotherapies are neuroprotective or prevent disease progression [179,180]. Similarly, no current therapy for Parkinson’s disease can delay progression, even if it manages symptoms [181]. Additionally, COMT-inhibitors and anticholinergics can result in significant differences in the gut microbiome [178,182] and have gastrointestinal side effects [183,184] that may be tied to gut dysbiosis. Thus, pharmacotherapies for neurodegenerative diseases currently available to manage symptoms are far from perfect given the side effects, the gut disruptive effects, and the lack of influence on disease progression. More research is required not only on the relationship between current pharmacotherapies and the gut microbiome and aging, but also on improving currently available pharmacotherapies to treat neurodegenerative diseases.

## 5. Future Directions

Although we have made great strides in the fields of microbiome research, as well as the study of aging, there is much left to do. With the multitude of interventions currently available and continually being investigated to promote healthy living and increase healthspan and lifespan, it is often difficult for individuals and their providers to determine which are best to utilize. Precision medicine offers one avenue to provide better management of care with the great individual variability in genes, environment, and lifestyle that can influence the outcome of many of the interventions that we touched upon in this review. For example, CR [159] and probiotics [185] can be impacted by sex and diet [186], and metformin [187] can be impacted by genetic differences. Precision medicine, and especially precision medicine involving the microbiome, is still a nascent field, but further development of the field would help in the treatment of age-related disease.

Furthermore, combination interventions may be useful for the treatment of some age-related disorders, such as AD [188]; however, more research needs to be done to determine the key factors to consider when to combine such interventions, such as when to begin the intervention [137]. For example, early in life may be an optimal time to target the gut microbiome with probiotics, as the neonatal microbiome is still forming and is thus more susceptible to change [189,190,191].

Research on the impact of cholinesterase inhibitors and memantine on the gut microbiome is important given the link between the gut microbiome and neurodegenerative diseases; further research may help us to elucidate how to mitigate the gut disruptive effects of these medications. For example, extensive research has shown how psychotropics are associated with gut dysbiosis, but probiotics, prebiotics, and fecal transplants may ameliorate this [192,193]. Therefore, promoting healthspan and lifespan is not only about adding more medications and interventions, but also about examining how to possibly reverse harm from other interventions.

In the future, it may also be possible to tailor and introduce diet modifications to facilitate gut microbiome-mediated improvements in healthspan and lifespan. It is important, however, to note that majority of the current mechanistic studies are limited to rodent or other animal models. The complexity and nature of interactions between the gut microbiome and the aging immune system could drastically vary between animal models and humans due to the presence of species-specific microbial populations [194,195]. It is therefore critical to increasingly conduct clinical studies and validate findings obtained from animal models in order to develop effective strategies to promote healthy aging in humans. As we look ahead at the fields of microbiome research and aging, the opportunities will continue to expand, and the connections among these physiological systems will continue to unfold.

## Data Availability

Not applicable.
